# The Inter-Relationship of Periostin, TGFβ, and BMP in Heart Valve Development and Valvular Heart Diseases

**DOI:** 10.1100/tsw.2011.132

**Published:** 2011-07-28

**Authors:** Simon J. Conway, Thomas Doetschman, Mohamad Azhar

**Affiliations:** ^1^ Riley Heart Research Center Wells Center for Pediatric Research Indiana University School of Medicine Indianapolis USA; ^2^ BIO5 Institute University of Arizona Tucson USA; ^3^ Department of Cellular and Molecular Medicine University of Arizona Tucson USA

**Keywords:** periostin, transforming growth factor beta, TGFβ, bone morphogenetic protein, BMP, heart development, heart valves, Marfan syndrome

## Abstract

Recent studies have suggested an important role for periostin and transforming growth factor *beta* (TGFβ) and bone morphogenetic protein (BMP) ligands in heart valve formation and valvular heart diseases. The function of these molecules in cardiovascular development has previously been individually reviewed, but their association has not been thoroughly examined. Here, we summarize the current understanding of the association between periostin and TGFβ and BMP ligands, and discuss the implications of this association in the context of the role of these molecules in heart valve development and valvular homeostasis. Information about hierarchal connections between periostin and TGFβ and BMP ligands in valvulogenesis will increase our understanding of the pathogenesis, progression, and medical treatment of human valve diseases.

## INTRODUCTION

About one-fourth of patients with congenital heart disease, which affects approximately 5% of live births, have structural anomalies of one or more heart valves[1,2]. Isolated bicuspid aortic valve and congenital polyvalvular disease represent major congenital cardiac valve malformations[3,4,5]. From a developmental point of view, the bicuspid aortic valve is usually not stenotic, but it may develop secondary stenosis or calcification. Bicuspid aortic valves with normal structure can be diagnosed under congenital polyvalvular disease. In congenital polyvalvular disease, two or more of the valves are congenitally abnormal (e.g., ventricular septal defect, tetralogy of Fallot), and show valve thickening and disrupted valve architecture. Congenital polyvalvular disease is typically seen in patients with trisomy 18, Noonan syndrome, and Marfan syndrome. In pediatric patients, 70–85% of stenotic aortic valves are bicuspid and at least 50% of adults with aortic stenosis have bicuspid aortic valves. If it is accepted that the incidence of bicuspid aortic valve is 1–2% and that serious complications occur in at least one-third of the cases, this condition may be responsible for more deaths and morbidity than the combined effects of all other congenital heart defects[6]. Interestingly, there is a marked distinction in the molecular profile between pediatric (i.e., stenotic) and adult (i.e., calcified) diseased aortic valves[7]. It has also been noted that aortic valves, and rarely tricuspid valves, develop calcification and that the pathology of diseased mitral valves includes myxomatous degeneration, but not calcification[8]. Myxomatous mitral valves are characterized by floppy leaflets and elongated or ruptured chordae tendineae, which have profoundly weakened material behavior compared to normal valve tissues[9]. Mature normal human valves have a trilaminar tissue organization of the extracellular matrix (ECM): elastin-rich atrialis (mitral/tricuspid valves) or ventricularis (semilunar valves), glycosaminoglycan (GAG)–rich spongiosa, and collagen-containing fibrosa[10]. Histologic findings of myxomatous valvular disease include highly fragmented collagen within the valve fibrosa and excess accumulation of GAGs, which causes billowing of the valve leaflets, with or without prolapse and regurgitation of mitral or aortic valves[11,12,13]. Regardless of the classification of valve disease, the valve thickening is often an early and major clinical complication of cardiac valve dysfunction in neonatal/infantile and adult patients with inherited (bicuspid aortic valve or congenital polyvalvular disease) or acquired conditions, including Marfan syndrome–like disorders, Noonan syndrome, isolated non–syndromic X-linked myxomatous valvular dystrophy, rheumatic arthritis, and anti–Parkinson's disease drug-induced (dopamine agonists, i.e., pergolide and cabergoline) valvular disease[14,15,16,17,18,19,20]. At the molecular and genetic levels, periostin and transforming growth factor *beta* (TGFβ) and bone morphogenetic protein (BMP) ligands have emerged as important players in heart valve formation and remodeling, and valvular heart diseases in mammals[21,22,23,24,25] (Fig. 1). In this review, we summarize the important current information about the individual activities of these molecules, and highlight their association and potential role in valve development and disease.

## FORMATION, REMODELING, AND MATURATION OF HEART VALVES

Cardiovascular development is considered a complex interplay of many cell-cell and cell-matrix interactions[26,27]. It is thought that disruption of *in utero* developmental processes in heart valve formation predispose patients to progressive postnatal valvular disease[22,28]. The process of valvulogenesis begins with an epithelial mesenchymal transition (EMT) (embryonic day [E] 9.5 to E10.5 in mice) that forms the endocardial cushions in the atrioventricular (AV) and conal outflow tract (OFT) regions of the developing heart[29,30]. Endocardial cushions are the primordia of valves and septa, and become mature structures through remodeling (E10.5–E18.5) and valve elongation and maturation (E14.5 to 1 week after birth). Heart valve remodeling can be conceptually organized into multiple aspects, including mesenchymal expansion (E10.5–E12.5), differentiation (E12.5–E16.5), and condensation (E15.5–E18.5), all of which occur in a temporally overlapping fashion[31]. It is very difficult to establish precisely when valvulogenesis formally ends. However, by systematic analysis of valve condensation, elongation, formation of nodular thickenings, and remodeling of tension-resistant ECM proteins in mouse valves from E15.5 to the 8-weeks-old stage, it has been demonstrated that valvulogenesis continues after birth to adjust to the postnatal maturation of the heart[32]. During the maturation process, the ECM of AV or OFT valves is organized into and maintained during adulthood as three distinct layers relative to blood flow: atrialis or ventricularis, spongiosa, and fibrosa[10].

## INVOLVMENT OF PERIOSTIN AND TGFβ SUPERFAMILY MEMBERS IN HEART VALVE DISEASES

Current evidence supports the involvement of periostin, TGFβs, and BMPs in both inherited congenital (bicuspid aortic valve and polyvalvular disease) and acquired valvular diseases affecting more than one valve type[21,25,33,34]. In mammals, the TGFβ subfamily has three members (TGFβ1, TGFβ2, and TGFβ3) and over 20 BMP members constitute the BMP subfamily[35,36]. Periostin is a 90-kDa secreted

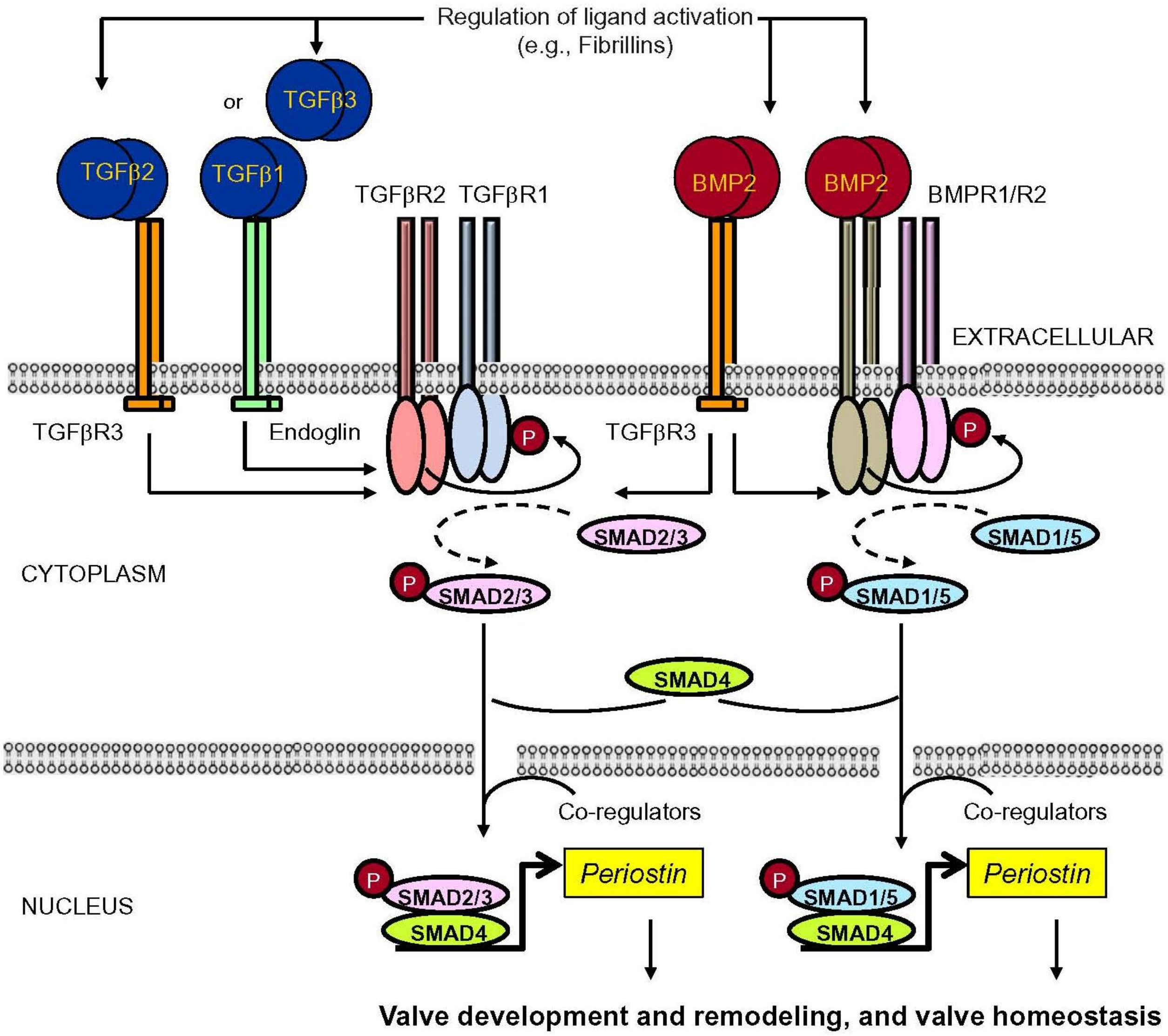

**FIGURE 1.** Schematic diagram illustrating the signaling events involving TGFβ, BMP, and perisotin. This association is based mostly on *in vitro* data and that this inter-relationship has not been completely proven *in vivo*. The major highlight of this illustration is that both TGFβ2 and BMP2 bind to TGFβR3, and signal through the canonical TGFβ signaling pathway during valve formation, which could affect periostin. ECM molecules, such as fibrillin-1, can restrict bioavailability of both TGFβ and BMP ligands. In the canonical TGFβ pathway, endoglin associates with TGFβ1 and TGFβ3 with a higher affinity than that of TGFβ2. TGFβR3 is a high-affinity receptor of TGFβ2. Both endoglin and TGFβR3 facilitates ligand binding to the canonical TGFβ-receptor (TGFβR1/TGFβR2) heteromeric complex. This results in phosphorylation of the TGFβ-specific SMADs (i.e., pSMAD2/3). The pSMAD2/3 forms a complex with SMAD4, which accumulates in the nucleus and can regulate *Postn*. Canonical BMP2 signaling occurs through BMP receptors and BMP-specific SMADs (i.e., SMAD1/5/8). BMP signaling has also been shown to induce *Postn*. Based on the individual activities of TGFβ, BMPs, and periostin in valvulogenesis, it is likely that this inter-relationship may play an important role in valvular heart diseases. See the text for more discussion.

protein that is involved in both valve development and valvular heart disease[21,37,38]. Periostin gene (*Postn*) knockout (hereafter termed *Postn*$^{-/-}$) mice have abnormal semilunar and AV valves, and periostin deficiency is associated to the stenotic bicuspid aortic valve in pediatric patients[21,39]. It is paradoxically elevated in diseased or thickened mitral valves in *Scleraxis* null mice[40]. TGFβ2 gene (*Tgfb2*) knockout (hereafter termed *Tgfb2*$^{-/-}$) mice have multiple structural defects, including thickened valves[41,42,43]. Genetic mutations in *FIBRILLIN-1* (and in the *Fibrillin-1*$^{+/C1039G}$ mouse model for Marfan syndrome), *TGFBR1/2*, *FILAMIN A* (encodes *SMAD2*-interacting protein), *SMAD3*, and *SMAD4* (common SMAD molecule for both TGFβ and BMP signaling), which are mediators of TGFβ and/or BMP signaling, are also associated with congenital valve diseases affecting inlet and/or outlet valves[17,19,33,34,44,45]. Many of these genetic mutations result in paradoxically increased TGFβ signaling[44,46,47]. Increased TGFβ1 and/or BMP2 signaling have also been reported in patients of valvular heart diseases, including chronic rheumatic heart disease and calcified human aortic valves[7,48,49]. Cardiac valve thickening is also associated with increased *Tgfb1* expression in response to serotonin agonists and to anti–Parkinson's disease drugs (dopamine agonists), such as pergolide and cabergoline[14,48]. The diagnosis of valvular heart diseases is typically done by echocardiography. There is no effective treatment to date to prevent the progressive changes in these valves and the patients often require surgery. The causes of various forms of valve diseases (i.e., stenotic/carcinoid/rheumatic/calcific) remain largely unclear. In this review, we have accumulated current evidence that suggests an association of periostin to TGFβ and BMP signaling in the pathogenesis of valve disease, which affects one or more valve types.

## ROLE OF PERIOSTIN IN VALVULOGENESIS

Periostin is a fasciclin domain–containing protein that plays important roles in the development and function of the cardiovascular system[37,38,50]. TGFβ-inducible protein (TGFβi/βigH3) and Stabilin-1 and Stabilin-2 are other similar proteins, and their role in cardiac development is discussed elsewhere[51]. As a secreted ECM protein that associates with areas of fibrosis, periostin can directly interact with other ECM proteins, such as fibronectin, tenascin-C, collagen I, collagen V, and heparin sulfate proteoglycans. Periostin serves as a ligand for select integrins, such as αvβ3, αvβ5, and α4β6, where it can affect the ability of cells (fibroblasts or cancer cells) to migrate and/or undergo mesenchymal transformation in select tissues[52,53,54]. Therefore, it is hypothesized that periostin can also signal through the integrin pathway during normal development and homeostasis. The mouse periostin gene (*Postn*) shows a dynamic expression profile, both developmentally and in adult tissues that are undergoing remodeling or active stress (reviewed[51]). Periostin is expressed in fibroblasts or in cells that adopt fibroblast-like characteristics following an injury event[21]. It has also been demonstrated that periostin is highly produced by cancer-associated fibroblasts or tumor stromal cells in various types of cancers, and in mature and developing heart valves[54,55]. Interestingly, periostin as well as TGFβ and BMP ligands are reported to be associated with cancer progression[56,57,58,59,60]. Periostin was first identified in osteoblast-like cells[61], and it is thought to play important roles in bone formation and development of periodontal ligaments[62,63]. Notably, periostin and TGFβ and BMP ligands are all involved in osteochondrogenesis[33,36,64]. Osteochondrogenesis has emerged as an important process in heart development and remodeling, and in the pathogenesis of valve disease[65,66]. The lesson learned from the many similarities between the roles of periostin and TGFβ and BMP signaling in cancer and bone/cartilage formation is that there may be an association between periostin and TGFβ and BMP signaling in the osteochondrogenic process that occurs during valvulogenesis.

In valvulogenesis, periostin is expressed in the differentiating cardiac cushion mesenchyme, but is absent from the cardiomyocyte lineage[21,55]. Although expression of the gene for TGFβi/βigH3 has some similarity with that of *Postn*, it is additionally expressed in the cushion myocardium in the outflow tract and is not expressed in AV cushions. On the other hand, Stabilin-1 gene expression is limited to the endocardium and has no overlap with *Postn*[51]. To investigate the role of periostin in heart valve development, we and others have generated mice that lack *Postn*[21,51]. The majority of *Postn*$^{-/-}$ mice survive, but they have abnormally shortened and thickened valve leaflets. A smaller percentage of these mice die before the weaning stage and valves in these mice are shorter than in the surviving *Postn*$^{-/-}$ mice. Both semilunar and AV valves are affected[21,39]. Adult *Postn*$^{-/-}$ mice show disrupted collagen matrix layers and higher than normal accumulation of valve matrix proteoglycans. In addition, the presence of ectopic cardiomyocytes and smooth muscles in valve leaflets is also noted in these mice. Some of these changes are consistent with the presence of a general valve pathology in these mice that can be associated with the onset and progression of mitral valve prolapse, aortic or pulmonary valve stenosis, and aortic regurgitation and calcification[21,22,28,67]. Overall, this suggests that loss of periostin results in inappropriate differentiation of mesenchymal cushions and in valvular abnormalities during establishment of valve leaflets.

## TGFβ AND BMP SIGNALING

There are 33 members in the mammalian TGFβ family, which include TGFβs, BMPs, activins and inhibins, nodal, myostatin, and the anti–Müllerian hormone[36]. TGFβ and BMP ligands are produced as large inactive complexes that are sequestered by a variety of ECM molecules, including fibronectin and fibrillins[36,68]. Current evidence suggests that the lack of fibrillin-1 could lead to ectopic activation of latent TGFβ and/or BMP ligands, which can then cause hyperactivated intracellular TGFβ and/or BMP signaling[69]. TGFβs usually interact with TGFβR2 and TGFβR1 (also called as ALK5) receptors[70], sometimes in conjunction with the type III TGFβ receptor (TGFβR3)[71] or endoglin[72], resulting in phosphorylation-dependent activation and nuclear localization of SMAD2/3 in association with SMAD4 (Fig. 1). TGFβ1 is also capable of signaling through ALK1 (a BMP type I receptor) in conjunction with endoglin in endothelial cells, thereby bringing about a cross-talk between ALK1- and ALK5-mediated TGFβ signaling[73,74]. Additionally, extracellular cues (e.g., fibronectin, periostin, and cell-cell contacts) acting through integrins can also affect TGFβ signaling[75]. TGFβs can also signal through several other pathways, such as the RAS-MAPK and retinoid pathways[70,76,77]. As with the TGFβs, the BMP subfamily of ligands also signals through a variety of type I and II receptors from which transduction occurs via SMADs 1, 5, and 8 in combination with SMAD4[36,78] (Fig. 1). The inhibitory SMAD6 predominantly inhibits BMP signaling, whereas SMAD7 inhibits both TGFβ and BMP signaling[79,80]. Recently, it has been shown that BMP2 can signal through TGFβR3, a canonical TGFβ receptor, in cardiac cushion EMT[81,82] (Fig. 1). Furthermore, ALK2, a BMP type I receptor, can participate in the TGFβ-dependent canonical SMAD2/3-mediated pathway[83]. Collectively, it appears that TGFβ and BMP ligands have both independent as well as overlapping activities during tissue development and homeostasis. In this review, we summarize some of these activities in the context of heart valve development and valvular heart disease (see below).

## ROLE OF TGFβ SIGNALING IN VALVULOGENESIS

Some clarity for TGFβ ligand specificity in heart valve development has been afforded by expression studies[23,84,85,86,87,88,89]. Briefly, *Tgfb2* is highly expressed during EMT (E9.5) within the cushion myocardium and its expression increases in the cushion mesenchyme during cushion remodeling (E12.5–E15.5). Intriguingly, TGFβ2 protein staining of the myocardium persists throughout development and in the adult, in the absence of detectable levels of the corresponding *Tgfb2* transcript[87]. *Tgfb1* is expressed predominantly in the endocardium (E9.5–onward) throughout development and neonates[84,86]. *Tgfb3* expression is limited to a subset of *Tgfb2*-expressing cushion mesenchymal cells and occurs only at later stages of cushion remodeling (E12.5–E16.5) during valvulogenesis[84,88]. Careful analysis of published expression data of *Tgfb1*-3 and *Postn* has also indicated that there is an overlap in the *Postn* expression with that of *Tgfb2* and *Tgfb3* during valvulogenesis[21,55,84]. Analysis of *Tgfb1-3* expression in adult mouse heart valves has not been done. Interestingly, there is significant *Postn* expression in postnatal valves, which is consistent with its important role in adult valve homeostasis[21].

Recently, serial analysis of gene expression (SAGE) profiling has been done to determine the genome-wide transcriptional patterns of the developing heart valves during EMT and cushion remodeling (E9.5–E12.5)[89]. SAGE permits the simultaneous evaluation of thousands of expressed transcripts, generating absolute values that are easily compared. For instance, SAGE data show that expression of *Tgfb2* is found to be high at E9.5–10.5 and then decreases over time, while *Tgfb3* expression is increased as valve development proceeds. While the *in vivo* roles of TGFβ1 and TGFβ3 in heart development remain unclear, this increased *Tgfb2* expression during the early heart development is consistent with the important role of TGFβ2 in the initiation and cessation of cushion EMT and cushion remodeling[42]. The fact that *Tgfb3* expression is on the rise as valve development proceeds into advanced stages of heart development suggests that TGFβ3 may play an important role in valve structure and function in postnatal mouse hearts. This speculation is consistent with the observations that genetic mutations or genetic polymorphisms in human *TGFB3* are involved in adult heart diseases[90,91]. Similarly, several studies have indicated an important role of periostin in adult heart diseases[51,92].

Although the functions of the TGFβ family members and receptors in EMT are well studied, their role in valve remodeling and maturation remains to be fully explored. There is some confusion about the role of TGFβ ligands in EMT during heart development or adult cardiac valves in mammals[30,93]. *In vitro* studies have suggested that while only TGFβ2 is required for EMT in mice and humans, addition of the other TGFβ ligands can also induce EMT in chickens and sheep in embryonic and adult hearts[23,85,93]. There is no report of significant cardiac developmental defects in TGFβ1- or TGFβ3-deficient mice[42,94,95]. On the other hand, consistent with the dominant expression in developing hearts, only *Tgfb2*$^{-/-}$ mice exhibit abnormal EMT, enlarged valves, and other congenital cardiovascular malformations[42,96]. Since gene expression data suggest that TGFβ2 and TGFβ3 could play roles in valve maturation within the valvular mesenchyme where *Postn* is also expressed, and since TGFβ1could be playing a juxtacrine/paracrine role from the valvular endocardium, further investigation is warranted to determine the functions of the TGFβs and their association with periostin in valvulogenesis.

## ROLE OF BMP SIGNALING IN VALVULOGENESIS

BMP2, 4, 5, 6, and 7 are the most studied members of the BMP subfamily of ligands in the cardiovascular system[97]. *Bmp2* is expressed in the AV myocardium during EMT, then switches to the cellularized cushion mesenchyme, where its expression continues throughout valvulogenesis[98,99]. *Bmp4* is expressed in both the AV and OFT myocardium[100]. *Bmp6* and *Bmp7* are coexpressed in the OFT myocardium, and *Bmp6* is uniquely expressed in the AV cushion mesenchyme, but not the OFT mesenchyme (reviewed[101]). With regard to the involvement of BMP signaling in heart valve disease, activated BMP signaling (as measured by pSMAD1/5/8) has been observed in diseased human aortic valves[7]. *Bmp2* is significantly expressed in adult mouse valves[102]. In humans, while the levels of BMP2 are significantly detectable in diseased valves, they are not detectable in normal human valves[49]. Moreover, BMP4 levels are highly abundant in adult cardiac valves[103]. This expression of BMP ligands in adult heart valves is consistent with the report signifying an *in vivo* genetic interaction between *Bmp2* and *Bmp4* as indicated by valve abnormalities in *Bmp2*$^{+/-}$*Bmp4*$^{+/-}$ adult mice[104]. Since periostin is also abundantly expressed in mature or adult valves[21], these findings suggest a coinciding expression of BMP4 and periostin in adult cardiac valves. These expression data also suggest that, to a large degree, BMP2 plays an important role in AV valve development; whereas BMP4, 6, and 7 are important for OFT valve development; and that BMP4 may be important for valve maturation and maintenance in adults.

Overall, as with some TGFβ ligands, there appears to be significant overlap in expression of specific BMP ligands and periostin during valvulogenesis and in adults.

At the functional level, the role of BMP ligands and BMP signaling in valvulogenesis is somewhat better understood than that of TGFβ ligands and TGFβ signaling. Here, we have summarized some significant studies highlighting the effect of loss-of-function and gain-in-function of BMP signaling on valve phenotype during heart development. A *Bmpr2* hypomorph has OFT defects, persistent truncas arteriosis, and hypoplastic semilunar valves, but no AV valve defects[105]. *Bmp6*$^{-/-}$ *Bmp7*$^{-/-}$ double knockout mice also have hypoplastic OFT cushions and semilunar valves[101]. Heart-specific (*Nkx2.5Cre*) conditional deletion of a floxed *Bmp2* allele leads to multiple defects in AV canal morphogenesis ranging from EMT to specification of the AV myocardium[98]. A similar approach to conditional mutation of *Bmp4* leads to OFT septation defects and hypoplastic OFT valves[100,106]. Intriguingly, conditional deletion of *Bmp4* in second heart-field lineage or endothelial-specific deletion of *Bmpr2* leads to hyperplastic or thickened valves[107,108]. It is noteworthy that gain of BMP function in *Noggin*$^{-/-}$ (a dedicated BMP antagonist) and *Smad6*$^{-/-}$ (Bmp-specific nuclear inhibitor) mice similarly results in thickened or hyperplastic valves[109,110]. Thus, these findings suggest that a genetic defect or loss-of-function in the BMP pathway activates major compensatory changes that culminate in a paradoxically excessive BMP and/or TGFβ response and valve thickening. These findings are in parallel to the studies indicating that both increased and decreased periostin and TGFβ signaling can cause valve thickening[17,21,40]. Overall, as with periostin and TGFβs, dysregulated BMP signaling can cause valve thickening.

## EVIDENCE OF THE INTER-RELATIONSHIP BETWEEN PERIOSTIN, TGFβ, AND BMP SIGNALING

Several recent studies have pointed to a cross-talk between TGFβ and BMP signaling that bears significant relevance to the inter-relationships between periostin, TGFβ, and BMP in valvulogenesis:

1. *Bmp2* is expressed in a similar fashion to *Tgfb2* in AV cushions during cushion formation in the mouse, and absence of *BMP2*, which results in down-regulation of *Tgfb2*, leads to complete shutdown of cushion EMT in mouse AV explant cultures[98,111]. This is consistent with the delayed augmentation of EMT *in vivo* in cushion formation in *Tgfb2*$^{-/-}$ mice[42].
2. TGFβR3, a high-affinity receptor of TGFβ, can bind with equal affinity to BMP2 and can lead to cushion EMT[81].
3. EMT as well as the phosphorylation of SMAD1/5/8 signaling can occur in the absence of endocardial-*Bmpr2*, indicating that BMP2 can signal through TGFβ or activin type II receptors[107].
4. TGFβ ligands can induce both TGFβ- and BMP-specific SMADs[83,112].
5. Endothelial-specific deletion of *Alk2*, a BMP type 1 receptor, leads to the down-regulation of both TGFβ- and BMP-specific SMADs[113].
6. *Postn* and *Tgfb* and *Bmp* ligand genes express in remodeling cushions, and periostin and *Tgfb2* are decreased in the absence of ALK3[114].

As BMP2 has also been shown to induce *Tgfb2*[98], and as BMP2 is known to function synergistically with *Tgfb3* in the regulation of endocardial cushion morphogenesis[115], it is possible that the effect of BMP signaling on *Postn*[21] is mediated by activating an endocardial TGFβ autocrine loop. Thus, it is currently not clear whether TGFβ2 and BMP2 share some common receptor(s) and SMAD(s) in cushion formation *in vivo*, and whether this cross-talk has relevance to periostin function in valvulogenesis.

## MOLECULAR REGULATION OF PERIOSTIN BY TGFβ AND BMP SIGNALING, AND ITS IMPLICATIONS IN VALVULOGENESIS

Despite the complex and intriguing correlation of deregulated *Postn* expression levels in both normal and pathological transformation conditions, very little is known about how *Postn* is transcriptionally controlled. Recently, a 3.9-kb$^{Postn}$ proximal promoter was isolated to understand the molecular regulation of *Postn*[116]. Although *Postn* is uniformly expressed in both AV and OFT cushions, the 3.9-kb$^{peri\text{-}lacZ}$ reporter expression is restricted to a subpopulation of distal OFT endocardial cushion cells. This indicates that elements required for AV cushion expression are absent from the 3.9-kb$^{Postn}$ promoter and may lie outside this 3.9-kb$^{Postn}$ domain. It is not clear whether the 3.9-kb$^{Postn}$ cell lineage can populate or colonize AV cushions later during valvulogenesis. In order to identify the enhancer and associated DNA binding factor(s) responsible for the restricted *Postn* expression, we identified a minimal 37-bp region within the 3.9-kb$^{Postn}$ enhancer that is a binding site to the ubiquitous *Ying Yang-1* (YY1) transcription factor. This YY1-binding was shown to be essential for *in vivo* 3.9-kb$^{peri\text{-}lacZ}$ promoter activity. Published data show that SMAD-mediated modulation of YY1 activity can regulate BMP responses and cardiac-specific expression of a GATA4/5/6-dependent *Nkx2.5* enhancer[117]. Thus, YY1 could be acting as a novel SMAD-interacting protein that represses SMAD transcriptional activities in a gene-specific manner and therefore regulates cell differentiation induced by TGFβ superfamily pathways[118]. Thus, whether TGFβ and/or BMP signaling regulates transcription of *Postn* via a direct regulation of the 3.9-kb$^{Postn}$ enhancer sequences should be explored.

Interesting correlations are observed between the mesenchymal gene expression patterns of *Tgfb2* and *Tgfb3* and *Postn*, and between membrane-restricted *Tgfb1* and *Stabilin1*, suggesting a functional interaction between fasciclin domain–containing proteins and specific TGFβ ligands[51,84]. *In vitro* studies have shown that *Postn* expression is positively regulated by TGFβ1[119,120]. Periostin levels are reduced in cardiac fibroblasts of *Tgfb2*$^{-/-}$ mouse embryonic hearts[50]. In chick AV cushions, TGFβ3 has been reported to induce periostin[121]. In addition, periostin is required for TGFβ signaling (i.e., pSMAD2) *in vivo* and TGFβ responsiveness *in vitro* in mouse embryonic fibroblasts (MEFs)[21]. Also, we reported that *Postn* is up-regulated in rat carotid arteries following balloon injury and in cultured vascular smooth muscle cells after stimulation with TGFβ1, and that up-regulation is mediated via PI3kinase[122].

There is circumstantial evidence that while TGFβ1 can induce *Postn in vitro* and under pathophysiological conditions[120], it is possible that TGFβ2 is the more likely inducer of *Postn in vivo* during valvulogenesis. Histological examination of hematoxylin and eosin (H&E)–stained serial sections showed that *Tgfb2*$^{-/-}$ mice, and not *Tgfb1*$^{-/-}$ and *Tgfb3*$^{-/-}$ mice, develop valve thickening as seen in *Postn*$^{-/-}$ mice[21,42]. It is currently not clear as to how TGFβ2 and periostin are connected. Since both the *Postn*-expressing 3.9-kb$^{peri\text{-}lacZ}$ cell lineage and cardiac neural crest cells (NCC) are predominantly restricted to the cardiac OFT[116,123,124], defects in one or both of these populations could result in abnormal OFT cushions in *Tgfb2* knockout mice. Through lineage tracking in *Wnt1Cre R26R Tgfb2*$^{-/-}$ embryos, no significant defect in the migration of cardiac NCC has been observed[125]. However, this does not rule out the possibility of an abnormal postmigratory NCC function in cardiac OFT cushions. On the other hand, valve maturation defects in both *Postn*$^{-/-}$ and *Tgfb2*$^{-/-}$ mice raise the possibility that there is a defect in *Postn*-expressing OFT cells in *Tgfb2*$^{-/-}$ mice. Another possibility is that there is an excessive amount of active TGFβ1 in *Tgfb2*$^{-/-}$ valves, which may increase periostin production and cause valve thickening, an association seen *in vitro* in cell culture (i.e., dermal fibroblast), and *in vivo* in mice and humans with heart diseases (see below)[17,21,92,120,126]. Collectively, these published findings lead to speculation that excessive amounts of active TGFβ1, hereinafter called “pathological TGFβ1” levels, may induce periostin and result in enlarged valves.

Disrupted ECM organization in *Postn*$^{-/-}$ mice is another mechanism by which excessive TGFβ1 activation and valve thickening can occur. Disrupted ECM leads to enhanced levels of active TGFβ1 (e.g., pSMAD2) and valve thickening in *Fibrillin1*-deficient mice[17,46]. It has also been shown that higher levels of TGFβ1 can lead to myofibroblast differentiation of valvular cells *in vitro*[127]. *Postn*$^{-/-}$ mice have aberrant valve muscularization, suggesting that loss of periostin results in inappropriate differentiation of valvular fibroblasts into myocardial or activated fibroblasts (i.e., myofibroblasts[21]). In avian models with experimentally induced cardiac abnormalities, it has been found that periostin is decreased, which correlates with valve thickening and persistent AV myocardial connections (e.g., Wolff-Parkinson White syndrome)[128]. The status of TGFβ signaling and its correlation to periostin and valve thickening are not determined in this avian model study. Consequently, we speculate that mouse models that produce pathological TGFβ1 during valvulogenesis will be useful in order to understand whether pathological TGFβ1 is sufficient for *Postn* and valve thickening in the absence of fibrosis. This will also illuminate the contribution of *Tgfb1* overexpression in several valvular diseases[46,48].

*Postn* expression is also induced *in vitro* by BMP signaling in cell culture experiments[129]. *Postn* is initially expressed at normal levels in E11.5 cushions of AV canal myocardium-specific conditional mutants of the BMP type 1 receptor (ALK3), but it is appreciably reduced in these mutant E14.5 AV leaflets[114]. Given that *Tgfb2* expression is also decreased in *Alk3*$^{-/-}$ mutants, the cardiac cushions of mutant mice may therefore be hypoplastic secondarily to decreased TGFβ2–mediated endocardial cushion EMT. This notion is also supported by our published findings indicating that TGFβ2 promotes early-stage EMT in the mouse[42]. In addition, gene-targeted deletion of *Postn* as well as *Smad6* or *Noggin* has revealed that both periostin and BMP signaling are required for normal valve formation and prevention of inappropriate differentiation along chondrogenic and bone lineages[21,109,110]. Our data have revealed that *Postn* is up-regulated in hyperplastic cushions of *Smad6*$^{-/-}$ hearts[21]. Thus, altered BMP signaling is upstream and may modulate *Postn* regulation, so that when the inhibitory SMAD or BMP antagonist are removed (leading to elevated BMP signaling), periostin is up-regulated. In support of this, exogenous chick *Bmp2* or constitutively active *Alk6* (type IB BMP receptor) retrovirus significantly promoted expression of *Postn* in AV cushion explant assays[130].

## PERSPECTIVES

The molecular regulation of *Postn* by TGFβ and BMP signaling as presented here is based on the association of these molecules and has not been proven (Fig. 2). Investigation into the regulation of *Postn* by TGFβ and BMP signaling during early development of OFT cushions and in the colonization of AV cushions would provide important information about the underlying mechanisms involved in valvulogenesis. Lineage analysis already demonstrates that the 3.9-kb$^{Postn}$ promoter marks a subpopulation of OFT cushion cells. It is unclear whether these 3.9-kb$^{Postn}$ marked cells can contribute to the AV valves during the later stages of valve development. It is unclear whether additional *cis*-elements that may lie outside the 3.9-kb$^{Postn}$ domain are necessary for the AV cushion recruitment of the 3.9-kb$^{Postn}$ marked cells. It remains to be determined if the AV cushion recruitment of the 3.9-kb$^{Postn}$–marked OFT cushion cells is TGFβ and/or BMP dependent. The 3.9-kb$^{Postn}$ enhancers can be utilized to drive novel tissue-specific expression of *Cre recombinase* in transgenic mice and to distinguish inlet *vs.* outflow cushion morphogenesis. Given the current lack of any robust cardiac mesenchyme–restricted promoters that work both in embryonic heart development and adult heart valves (in valve interstitial cells and cardiac fibroblasts), 3.9-kb$^{Postn}$-Cre driver mouse lines may provide useful tool mice for the scientific community interested in cardiac development and disease.

It is noteworthy that periostin is found in osteoblast-like cells and that it is dysregulated in various forms of cancer. The processes of valvulogenesis, osteogenic and chondrogenic development, and tumorigenesis have many features in common. For instance, periostin and TGFβs and BMPs are all reported to be associated with cancer progression and osteochondrogenic processes (e.g., calcification of bones and valves). Since both TGFβs and BMPs play important roles in similar processes and their roles in these processes are currently better understood than that of periostin, the information gained from these other fields of study should provide additional clues about periostin function and its regulation during

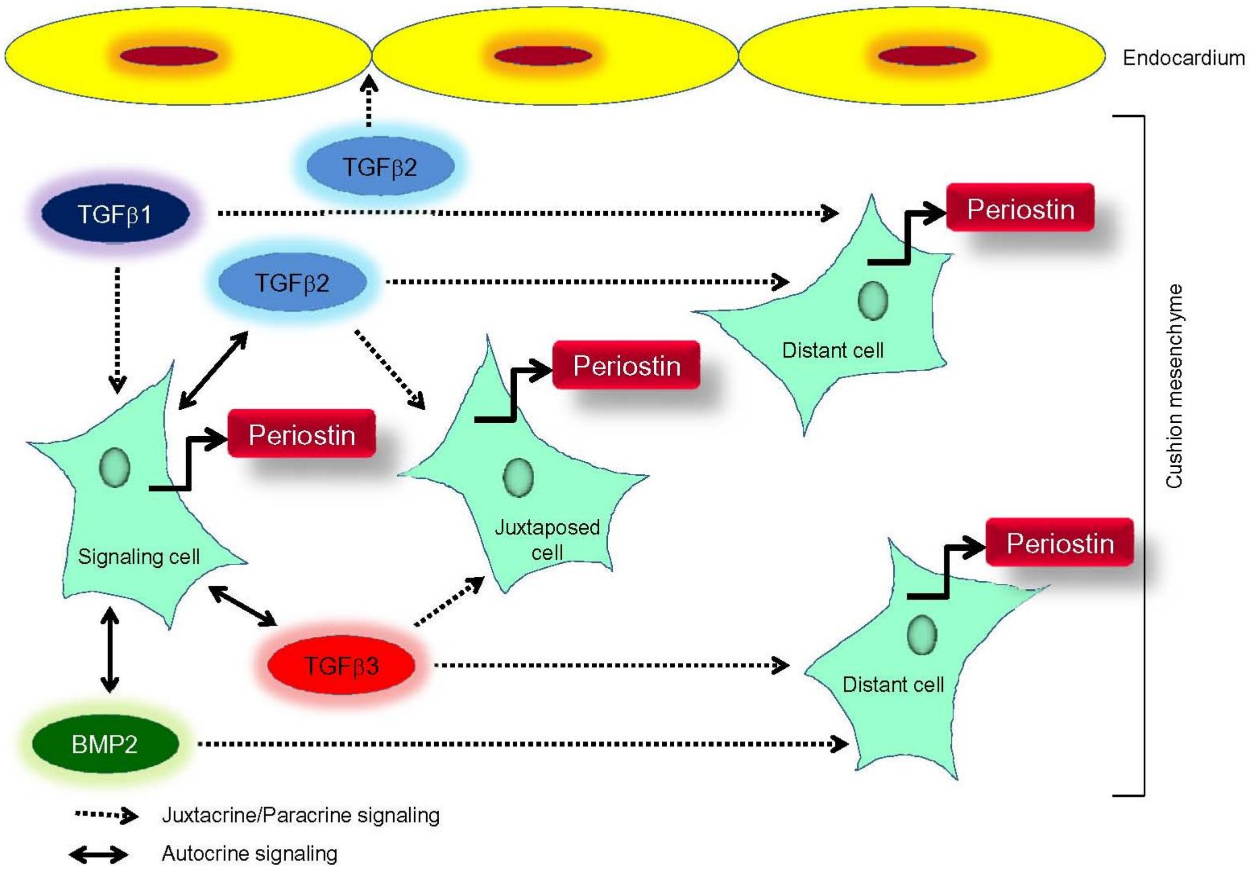

**FIGURE 2.** The inter-relationship of periostin, TGFβs, and BMP2 in AV cushion remodeling during heart development. In this model, periostin is tied up to a network of TGFβ and BMP signaling, which is required for various aspects of valvulogenesis, such as matrix organization, suppression of nonvalvular phenotypes (i.e., ectopic presence of bone and cartilage lineage) and valve differentiation, and valve structure and function in adult hearts (see the text for details). Depiction of the cell type–specific patterns of expression of TGFβ and BMP ligand genes and periostin during cushion remodeling is based on published data. Speculation about the role of cell autonomous and nonautonomous action of the TGFβ and BMP signaling network on perisotin is also based on published information. Briefly, TGFβ2, TGFβ3, and BMP2 are predominantly produced in the cushion mesenchyme, where they could engage in autocrine (double-headed arrows) and juxtacrine and paracrine (dotted arrows) signaling to induce *Postn* during cushion remodeling. Autocrine signaling shows that TGFβ or BMP ligands are secreted or produced by and act on the same cell. The term juxtacrine signaling is used here to highlight the action of the secreted TGFβ or BMP ligands on the adjacent cell. Paracrine signaling refers to the action of the secreted TGFβ or BMP ligands on a distant cell population in the remodeling cushions. Cushion mesenchymal TGFβ2 has been shown to have an important paracrine/juxtacrine role in the cessation of cushion EMT. TGFβ1 is primarily seen in the cushion endocardium during heart development. Since it can induce *Postn* in different cell types *in vitro*, it is possible that TGFβ1 could have a paracrine/juxtacrine effect on periostin in the cushion mesenchyme. Consequently, it appears that the cushion mesenchyme have various contingent sources and mechanisms to produce and sustain periostin during valvulogenesis. The model presented here on the molecular regulation of periostin by TGFβ1-3 and BMP2 in the context of valvulogenesis is predominantly based on the published association of these molecules and is not proven (see the text for further details).

valvulogenesis. Several studies that are summarized here have called for an investigation to delineate the relationship between periostin and TGFβ and BMP signaling, and how these molecules influence valve thickening (Fig. 2). In summary, clarifying the inter-relationship between periostin, TGFβ, and BMP during heart development and in adult valve homeostasis will lead to a better understanding about the pathogenesis of valvular heart diseases.

## ACKNOWLEDGMENTS

This work was supported, in part, by the Indiana University Department of Pediatrics (Cardiology), Riley Children's Foundation, BIO5 Institute (University of Arizona), Arizona Biomedical Research Commission Project #0901, and the National Institutes of Health (NIH) grants R01 HL60714, P01 HL85098, R01 HL092508, HL070174, HL105280.
